# Abacus in the Brain: A Longitudinal Functional MRI Study of a Skilled Abacus User with a Right Hemispheric Lesion

**DOI:** 10.3389/fpsyg.2012.00315

**Published:** 2012-08-28

**Authors:** Satoshi Tanaka, Keiko Seki, Takashi Hanakawa, Madoka Harada, Sho K. Sugawara, Norihiro Sadato, Katsumi Watanabe, Manabu Honda

**Affiliations:** ^1^Center for Fostering Young and Innovative Researchers, Nagoya Institute of TechnologyNagoya, Japan; ^2^Graduate School of Health Sciences, Kobe UniversityKobe, Japan; ^3^Department of Functional Brain Research, National Institute of NeuroscienceKodaira, Japan; ^4^Department of Rehabilitation Medicine, Eisei HospitalHachioji, Japan; ^5^Division of Cerebral Integration, National Institute for Physiological SciencesOkazaki, Japan; ^6^School of Life Sciences, The Graduate University for Advanced Studies (SOKENDAI)Hayama, Japan; ^7^Research Center for Advanced Technology and Science, The University of TokyoKomaba, Japan

**Keywords:** acalculia, arithmetic, calculation, expertise, imagery, memory, plasticity, stroke

## Abstract

The abacus, a traditional physical calculation device, is still widely used in Asian countries. Previous behavioral work has shown that skilled abacus users perform rapid and precise mental arithmetic by manipulating a mental representation of an abacus, which is based on visual imagery. However, its neurophysiological basis remains unclear. Here, we report the case of a patient who was a good abacus user, but transiently lost her “mental abacus” and superior arithmetic performance after a stroke owing to a right hemispheric lesion including the dorsal premotor cortex (PMd) and inferior parietal lobule (IPL). Functional magnetic resonance imaging experiments were conducted 6 and 13 months after her stroke. In the mental calculation task, her brain activity was shifted from the language-related areas, including Broca’s area and the left dorsolateral prefrontal and IPLs, to the visuospatial-related brain areas including the left superior parietal lobule (SPL), according to the recovery of her arithmetic abilities. In the digit memory task, activities in the bilateral SPL, and right visual association cortex were also observed after recovery. The shift of brain activities was consistent with her subjective report that she was able to shift the calculation strategy from linguistic to visuospatial as her mental abacus became stable again. In a behavioral experiment using an interference paradigm, a visual presentation of an abacus picture, but not a human face picture, interfered with the performance of her digit memory, confirming her use of the mental abacus after recovery. This is the first case report on the impairment of the mental abacus by a brain lesion and on recovery-related brain activity. We named this rare case “abacus-based acalculia.” Together with previous neuroimaging studies, the present result suggests an important role for the PMd and parietal cortex in the superior arithmetic ability of abacus users.

## Introduction

**Figure 1:**
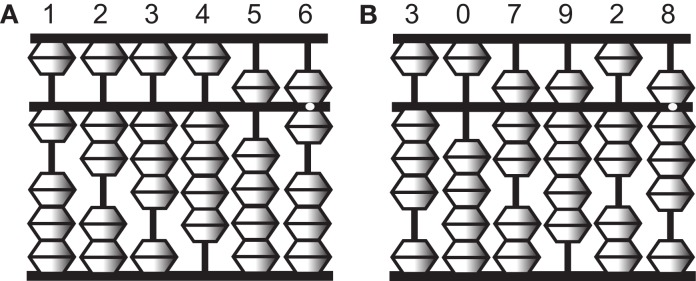
**Illustrations of an abacus**. An abacus is a rectangular wooden calculator based on the decimal system. Each vertical rod has five sliding beads, one above and four below a middle horizontal bar. Numbers are represented by the configurations of the beads. A bead above the bar is equal to five when it is pushed down, and each of the four beads below is equal to one when pushed up. For example, the left figure **(A)** represents 123,456 and the right figure **(B)** represents 307,928.

To perform complex calculations, most people rely on physical devices such as pencil and paper, mechanical calculators, and more recently digital computers. One such device is an abacus, which is still widely used in Asian countries. The abacus is a simple device of beads and rods, and numbers are represented by the spatial locations of beads (Figure 1). Skilled abacus users can calculate accurate answers to mathematical problems extremely rapidly. Interestingly, however, abacus users not only manipulate the tool skillfully in its physical form but also gain the ability to mentally calculate extraordinarily large numbers, often more than 10 digits at the expert level, with unusual speed and accuracy (Hatano et al., 1977). Psychological studies have shown that a non-linguistic strategy using visual imagery of the abacus (a “mental abacus”) underlies this unusual calculation ability (Hatano et al., 1977, 1987; Hatano and Osawa, 1983; Stigler, 1984; Hatta et al., 1989; Hishitani, 1990; Hanakawa et al., 2004; Tanaka et al., 2008; Frank and Barner, 2012). These works have demonstrated examples of the role of mental imagery in mental arithmetic operations.

Several behavioral and neuroimaging studies have attempted to examine the neural correlates of the calculation strategy employed by abacus users (Hatta and Ikeda, 1988; Tanaka et al., 2002, 2008; Hanakawa et al., 2003; Chen et al., 2006; Wu et al., 2009; Hu et al., 2011; Ku et al., 2012). For example, recent neuroimaging studies have reported activation in the bilateral dorsal premotor cortex (PMd) and inferior and superior parietal lobule (IPL and SPL, respectively) during mental calculation and digit memory tasks in abacus users (Tanaka et al., 2002; Hanakawa et al., 2003; Chen et al., 2006; Wu et al., 2009; Ku et al., 2012). However, there have been no neuropsychological studies that report deficits in mental abacus ability after focal brain injury. Therefore, the causal relationship between mental abacus ability and region-specific brain structures remains unclear.

Here, we report the case of a patient who was a well-experienced abacus user but had impaired mental arithmetic performance based on her mental abacus strategy due to a stroke. Her knowledge of basic arithmetic facts and her knowledge and operation of a physical abacus were intact. Only performance in mental calculation and digit memory tasks based on the mental abacus strategy was transiently impaired after the lesion. When we met her for the first time, she said “I lost my abacus in the brain.”

The first purpose of the present study was to localize the lesion areas using high-resolution structural magnetic resonance imaging (MRI) with a 3T MRI scanner. We hypothesized that the lesion areas should include the PMd and/or parietal regions that were dominantly activated during the mental calculation and digit memory tasks in the previous functional MRI studies of abacus users (Tanaka et al., 2002; Hanakawa et al., 2003; Chen et al., 2006; Ku et al., 2012).

The second purpose of the present study was to examine the changes of brain activity with the recovery of mental abacus ability. Several neuroimaging studies have reported changes of brain activities with recovery from motor, attentional, or language deficits after stroke (Ward et al., 2003; Fridman et al., 2004; Corbetta et al., 2005; Price and Crinion, 2005; Heiss and Thiel, 2006). However, recovery-related changes in brain activity from deficits in arithmetic ability, especially in the non-linguistic aspects of arithmetic operation, remain totally unknown.

We hypothesized that the patient would change her strategy for mental calculation and digit memory from verbal to visuospatial with stroke recovery. Therefore, her brain activity during mental calculation would shift from language-related to visuospatial-related brain regions after recovery. As mentioned above, previous imaging studies have revealed dominant activation in the bilateral PMd, IPL, and SPL during mental calculation in abacus experts (Tanaka et al., 2002; Hanakawa et al., 2003). Neuroanatomical studies have shown that the PMd and parietal cortex have dense neuroanatomical connections (Wise et al., 1997; Luppino et al., 1999; Wise and Murray, 2000). Thus, the PMd, IPL, and SPL may work as a functional network during abacus-based mental calculation. Damage in one node may induce transient impairment of mental abacus ability. However, it is possible that the other intact nodes in the functional network could gain the ability to work without the damaged node, possibly because of functional reorganization within the remote intact nodes (Frost et al., 2003; Fridman et al., 2004; Dancause et al., 2006). Thus, we hypothesized that the intact PMd, IPL, and/or SPL would be active with the recovery of mental abacus ability.

In the present study, functional MRI experiments were conducted 6 and 13 months after her stroke and brain activity between the two sessions was compared in order to test this hypothesis. In addition, a behavioral experiment using dual-task interference paradigms was conducted to confirm her use of the mental imagery of an abacus on a digit memory task 13 months after her stroke.

## Materials and Methods

### Case report

The patient was a 57-year old left handed female. She had worked as a professor in a national university before the stroke. She had a Ph.D. degree in medicine and had worked as a scientist in the field of neuropsychology for more than 25 years. She had published more than 20 international peer-reviewed papers. She had also engaged in rehabilitative medicine as a speech-language-hearing therapist for more than 25 years.

She started her abacus training at an abacus school when she was an elementary-school child, and had trained in physical and mental abacus operation for 3 years. We speculated that she was an excellent and skilled abacus user owing to the fact that she became a finalist at a domestic abacus competition in Japan in two successive years, although her training period was relatively shorter compared with the grand experts who participated in our previous functional MRI studies (Tanaka et al., 2002; Hanakawa et al., 2003). After she finished her abacus training, she kept using abacus-based mental calculation and mnemonic strategies in everyday activities for a long period and did not lose her ability. In fact, she reported that her forward digit span was around 12 before the stroke episode. This was far beyond the average score for her age group.

In July 2009, she suffered from a right hemispheric infarct in the territory of the anterior and middle cerebral arteries. When a therapist tested her digit span during a clinical neuropsychological evaluation in a hospital approximately 2 months after her stroke, she noticed that she was not able to use the mental abacus strategy for the digit span test. She was not able to generate vivid mental imagery of an abacus and the image of the abacus was very fragile. Detailed structural MRI scans were obtained in January 2010. Functional MRI scans were conducted at two different periods, the first in January 2010 and the second in August 2010.

### Neuropsychological evaluation

Neuropsychological evaluations were conducted approximately 1 month after stroke onset. Her score on Raven’s Standard Progressive Matrices was in the average range (33/36). Similarly, her IQ measured by Kohs Block Design Test was also in the average range (108). The Standard Language Test for Aphasia (SLTA; Hasegawa et al., 1984), which has been widely used in Japan, did not detect any impairments of language. However, clinical observation detected mild impairments of her speech production: her prosody was impaired and speed of speech was slow with small volume. Clinical observation immediately after her stroke detected unilateral visual neglect. For motor function, the patient showed a severe paralysis in the left upper limb and mild paralysis in the left lower limb.

### Arithmetic ability

After her stroke onset, her arithmetic ability was not impaired according to the neuropsychological evaluation. She was able to perform four basic arithmetic operations without any problem. In fact, she was able to answer all arithmetic problems correctly in the SLTA. In addition, her long-term memory of digits was also intact because she correctly remembered the numbers of her bank accounts and airplane mileage accounts. However, she noticed that she was not able to generate visual imagery of a mental abacus, which had been easily generated before the stroke, when a neuropsychologist tested her maximum digit span 2 months after her stroke. Before the stroke, she used to use the mental abacus strategy especially when she calculated and memorized larger sequences of digits, because the visuospatial strategy, rather than a phonological strategy, was useful in coding a larger number of digits (Hatano et al., 1977; Hatano and Osawa, 1983). Due to the impairment of visual imagery after her stroke, she used the phonological strategy instead. She was able to perform four basic arithmetic operations correctly although she felt that her arithmetic ability had declined after her stroke.

Six months after her stroke, just before the first functional MRI session, we evaluated her knowledge of basic arithmetic facts, as well as her knowledge, and operation of a physical abacus. These aspects were all intact. However, she still felt that it was difficult to generate a vivid visual image of a mental abacus. She reported that she was not able to perform mental calculations and memorize digit sequences based on the mental abacus strategy because her mental abacus was fragile. However, 13 months after her stroke, she reported that her capacity for visual imagery of a mental abacus had recovered. At that time, she participated in the second functional MRI session.

**Figure 2:**
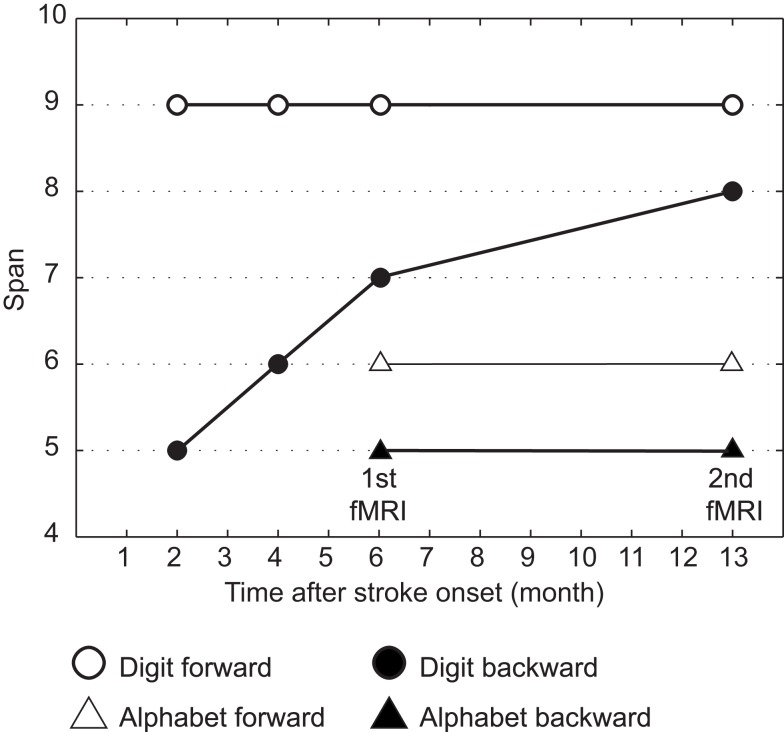
**Behavioral performance of maximum digit and alphabet span tasks**. Maximum digit spans were higher compared with alphabet span, indicating that her superior performance was digit memory-specific. Her maximum forward digit span (white circle), as well as forward and backward alphabet spans (white and black triangles), was unchanged across the entire experimental period. However, the maximum backward digit span (black circle) was improved over time after her stroke.

Figure 2 shows her behavioral performance of maximum digit and alphabet span tasks. Forward digit span and forward and backward alphabet spans were all unchanged across the experimental period. In contrast, backward digit span improved over time after her stroke. Her backward digit span 13 months after her stroke was eight and almost equal to her forward digit span. It has been reported that abacus experts reproduce a series of digits in backward order almost as well as in the forward order, because both require experts to read off the digits from visuospatial mental representation of an abacus (Hatano and Osawa, 1983). Therefore, nearly identical maximum digit spans both backward and forward might be interpreted as evidence that she used her mental abacus 13 months after her stroke. In fact, she reported that she was able to use the mental abacus strategy for the backward digit span task 13 months after her stroke.

### Experimental procedure

The patient gave written, informed consent before the experiments, which were approved by the local ethics committee of the National Institute for Neuroscience.

The patient participated in two functional MRI sessions of the mental calculation and digit memory tasks (Experiment 1). The first and second functional MRI sessions were conducted 6 months (January 2010) and 13 months (August 2010) after her stroke onset, respectively. The difference of the brain activities between the two sessions was compared. Structural MRI scans were obtained in January 2010. In addition, the patient participated in a behavioral experiment after the second functional MRI session in order to examine whether the patient would use abacus-based mental calculation and digit memory strategies in these tasks (Experiment 2).

### Experiment 1

#### Behavioral task in functional MRI experiment

For the functional MRI experiment, the patient performed mental calculation and digit memory tasks that were used in our previous functional MRI studies of abacus experts (Tanaka et al., 2002; Hanakawa et al., 2003). Before the functional MRI experiment, she practiced these tasks outside the scanner to become familiar with the tasks. Presentation software (Neurobehavioral Systems Inc., Albany, CA, USA) was used for the visual stimulus presentation and to record her responses. Stimuli were presented on a screen using a liquid crystal display projector, and she viewed the screen though a mirror.

**Figure 3:**
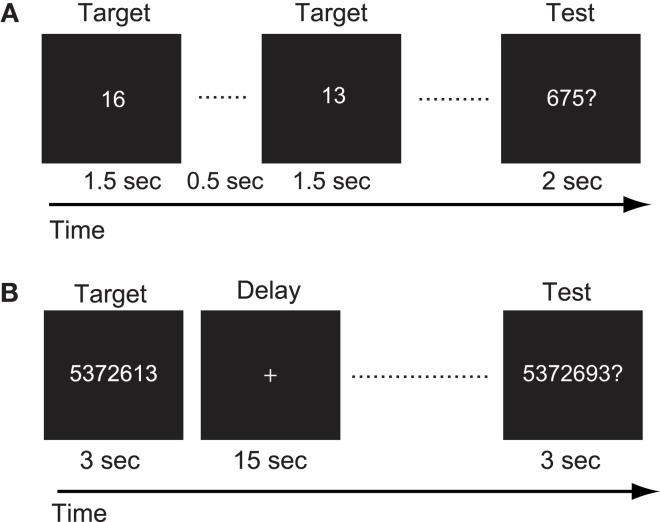
**(A)** Schematic illustration of the mental calculation task. The patient was asked to add a series of numbers mentally that were visually presented on the computer screen. **(B)** Schematic illustration of the digit memory task. She was asked to retain the target sequence of digits during the delay period.

For the mental calculation task, white digit stimuli were presented for 1.5 s with inter-stimulus intervals of 2 s on the center of a screen (Figure 3A, Hanakawa et al., 2003). Digit stimuli were presented 10 times during each trial. The patient was asked to mentally add the presented series of digits without moving her fingers. After the presentation of these digit stimuli, a red digit stimulus was presented for 3 s. She was asked to judge whether the addition answer in her mind and the test digit stimuli were the same or different, by pressing one of the response buttons with the right fingers. After each trial, there was an 18-s inter-trial interval (ITI) in which the patient simply watched the white fixation cross presented at the center of the screen (visual fixation condition). She performed additional tasks with single-digit and two-digit numbers. The experimental session consisted of five trials for each task in an alternate order.

For the digit memory task, a delayed match-to-sample task using a digit sequence as the stimulus was employed (Figure 3B, Tanaka et al., 2002). A target digit sequence was presented on the center of a screen for 3 s. The length of the digit sequence was a five digit number, which was two digits shorter than her digit span memory capacity measured before the first functional MRI session. After a 15-s delay period, during which only a fixation cross appeared on the screen, a test sequence of digits was presented for 3 s. She was asked to judge whether the target and test sequences were the same or different, by pressing one of the response buttons. Following these behavioral events, there was a 17-s visual fixation. The experimental session consisted of 10 trials.

The patient also participated in functional MRI experiments of verbal fluency and hand grip tasks 13 months after the stroke. These experiments were conducted to ascertain whether the region-specific brain activity during arithmetic tasks 13 months after the stroke would be task-specific or not. In the verbal fluency task, the subject was asked to generate in her mind as many words as possible from an indicated category (such as names of sports or fruits) during a 24-s trial. After each trial, there was a 24-s visual fixation condition. The task and fixation condition were alternately performed 10 times. In the hand grip task, the patient was asked to make the hand grip movement with her paretic hand every 2 s during a 24-s period. The hand grip task and visual fixation condition were alternately performed 10 times.

#### Imaging data acquisition and analysis

The functional MRI experiment was conducted using a 3.0-T MRI scanner (MAGNETOM Trio, Siemens, Erlangen, Germany). Functional images were acquired using a T2^*^-weighted echo planar imaging sequence (TR/TE/FA/FOV/voxel size/slice number = 3000 ms/30 ms/90°/192 mm/3.0 mm × 3.0 mm × 3.0 mm/46 axial slices for the mental calculation task, and 2000 ms/40 ms/80°/192 mm/3.0 mm × 3.0 mm × 4.0 mm/25 axial for the digit memory task). A total of 143 and 205 functional images on each mental calculation and digit memory task were collected during each session. The first three and five images of each task were discarded from data analysis to allow for the stabilization of the magnetization. Eighty-three images were obtained on each verbal fluency and hand grip task and the first three images were discarded. A high-resolution structural T1 image was acquired using a Magnetization Prepared Rapid Acquisition in Gradient Echo (MPRAGE) sequence.

SPM8 software (Wellcome Department of Cognitive Neurology, London, UK) was used for image processing and analysis. The T1 image was spatially normalized to fit a Montreal Neurological Institute (MNI) template (Evans et al., 1993). The damaged regions were masked to reduce the influence from non-brain or lesioned tissue (Brett et al., 2001). For functional images, the data were first realigned to the mean functional images in order to reduce the effect of head motion. These images were then normalized to the MNI template, with the same parameter obtained for T1 normalization. Then, the images were spatially smoothed using an isotropic Gaussian kernel of 6-mm full-width half maximum (FWHM).

#### Statistical analysis

Statistical analysis of the time course data at each voxel was conducted with a general linear model in order to identify voxels that showed task-specific and session-specific signal changes (Friston et al., 1994). The brain activities in the mental calculation and digit memory tasks were analyzed separately.

For the mental calculation task, one-digit and two-digit calculation tasks were separately modeled as regressors on each session with boxcar functions convolved with a hemodynamic response function. For the digit memory task, the presentations of the target and test sequences, and the delay period, were separately modeled on each session using three boxcar functions convolved with a hemodynamic response function. For the verbal fluency and hand grip task, the task period was modeled using three boxcar functions convolved with a hemodynamic response function. In all tasks, head-movement parameters were also included as regressors of no interest.

To test hypotheses about regionally specific task-effects or session-effects, the estimates for each model parameter were compared with the linear contrasts. The resulting set of voxel values constituted a statistical parametric map of the *t* statistic, SPM{*t*}. In all tasks, the statistical threshold was set at *p* < 0.001 at the voxel level. Control for multiple comparisons was achieved at the cluster level with Gaussian random field theory either in the whole brain (*p* corr < 0.05) or the small volume around the coordinates of the regions of interest (ROIs) based on the published papers (*p* svc < 0.05). On the basis of previous works on abacus experts (Tanaka et al., 2002; Hanakawa et al., 2003), spherical ROIs (*r* = 8 mm) were created at the peak voxel in the bilateral SPL (left *x* = −18, *y* = −66, *z* = 60; right *x* = 14, *y* = −66, *z* = 64 at MNI coordinate), left IPL (*x* = −46, *y* = −40, *z* = 54), left PMd (*x* = −32, *y* = −6, *z* = 52), and Broca’s area (*x* = −50, *y* = 10, *z* = 26).

### Experiment 2

#### Behavioral evaluation in mental abacus use

A behavioral experiment using interference paradigms was conducted to examine whether the patient would utilize the mental abacus strategy on a digit memory task 13 months after her stroke (Figure 10A). The behavioral paradigm was based on Hatta et al. (1989). She performed a delayed digit recall task. First, a target digit sequence was presented on the computer screen for 3 s. The length of the target digit sequence was eight, which was one-digit shorter than her maximum digit span memory capacity. After a 15-s retention interval, she was asked to recall and report the digit sequence orally. There were three experimental conditions which differed according to the types of visual distractors. Pictures of abacus figures, human faces, or gray rectangles were presented on the center of the screen during the retention interval. Each distractor stimulus was presented for 1 s with 0.5 s inter-stimulus intervals. She performed 15 trials for each distractor condition. We hypothesized that if she utilized a mental abacus for the digit memory task, the presentation of the pictures of abacus figures would interfere with task performance more than the presentation of the human faces and gray rectangles.

## Results

### Structural MRI

**Figure 4:**
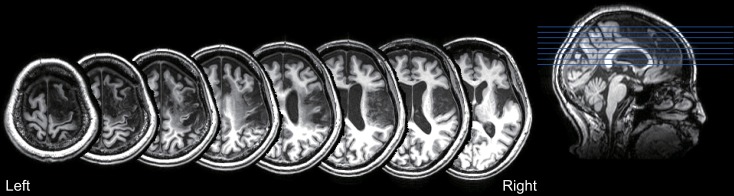
**T1-weighted structural MRI of the patient**. The lesion was observed in the fronto-parietal cortex, including the posterior parts of the inferior and superior frontal gyrus, anterior insula, anterior cingulate gyrus, pre and post central gyrus, and supramarginal gyrus. No lesion was observed in the left hemisphere.

The T1-weighted MRI showed a right fronto-parietal lesion, involving the posterior parts of the inferior and superior frontal gyrus, anterior insula, anterior cingulate gyrus, pre and post central gyrus, and supramarginal gyrus (Figure 4). These lesioned areas included the right PMd and IPL, which were dominantly activated during the mental calculation and digit memory tasks in the previous functional MRI studies of abacus experts (Tanaka et al., 2002; Hanakawa et al., 2003; Chen et al., 2006; Ku et al., 2012). The lesion was not observed in the left hemisphere.

### Experiment 1: Functional MRI experiment

#### Mental calculation task

**Figure 5:**
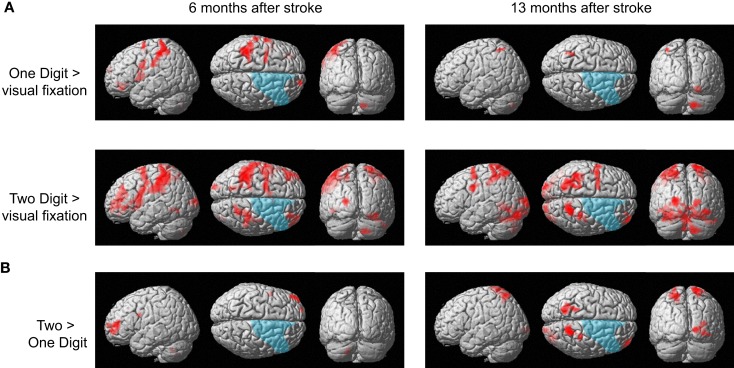
**(A)** Active areas during the mental calculation task relative to those during the visual fixation condition 6 and 13 months after her stroke. **(B)** Active areas during the two-digit mental calculation task relative to those during the one-digit task 6 and 13 months after her stroke. The blue region indicates the lesion area. Brain activity is overlayed on the spatially normalized rendering images (voxel level, *p* < 0.001 uncorrected for multiple comparison; cluster level *p* < 0.05 FWE). The detailed coordinates and statistical values are listed in Table A1 in Appendix online.

The patient responded correctly in all trials of the calculation tasks in both functional MRI sessions. Figure 5A shows brain activity associated with one- and two-digit mental calculation tasks relative to the visual fixation condition (see Table A1 in Appendix online). In one-digit mental calculations, brain activity was generally lateralized to the left hemisphere both 6 and 13 months after her stroke. In contrast, brain activity in two-digit mental calculations was observed bilaterally both 6 and 13 months after her stroke. These brain regions include the middle frontal gyrus, pre- and postcentral gyrus, SPL, middle and superior occipital gyrus, inferior temporal gyrus, and cerebellum. This activity was not observed in the damaged regions of the right hemisphere. When brain activities during one- and two-digit mental calculation tasks were directly compared, significant brain activity in the left middle frontal gyrus was observed 6 months after her stroke (Figure 5B). In contrast, significant activity was observed in the bilateral SPL, right middle frontal gyrus, postcentral gyrus, and middle occipital gyrus 13 months after her stroke.

To investigate the time-specific brain activities, her whole brain activities between 6 and 13 months after her stroke were directly compared. A previous study has revealed that the region-specific brain activities in abacus users were more evident in the mental calculation task with a higher cognitive demand (Hanakawa et al., 2003). Therefore, the brain activities in the two-digit addition task between 6 and 13 months after her stroke were compared in the analysis.

**Figure 6:**
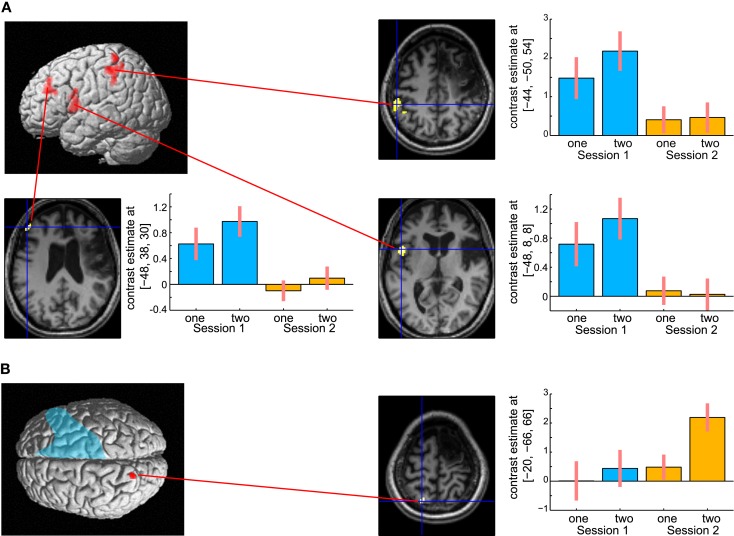
**(A)** Active areas during the mental calculation task-specific to 6 months after her stroke. These areas include Broca’s area, the left dorsolateral prefrontal cortex and the inferior parietal cortex. **(B)** Active areas specific to 13 months after her stroke. Only the left superior parietal cortex was significantly activated on this comparison. The blue color on the rendering structural image indicates the damaged cortical area. Only statistically significantly different activity is shown (voxel level, *p* < 0.001 uncorrected for multiple comparison; cluster level *p* < 0.05 FWE). In each bar graph, the vertical axis indicates contrast estimated values relative to the baseline, whereas the horizontal axis indicates the type of task. One and two in the figure indicates one-digit and two-digit addition task, respectively. Blue and orange color bar indicates session 1 and 2, respectively. The error bars represent standard deviations across scans.

The results are shown in Figure 6. The left hemispheric cortical activities including Broca’s area (peak coordinate *x* = −48, *y* = 8, *z* = 8; *t* = 4.73, cluster size = 227 voxels, *p* corr < 0.05), the left dorsolateral prefrontal cortex (DLPFC, *x* = −48, *y* = 38, *z* = 30; *t* = 4.81, cluster size = 118 voxels, *p* corr < 0.05), and IPL (*x* = −44, *y* = −50, *z* = 54; *t* = 4.38, cluster size = 118 voxels, *p* corr < 0.05) were significantly greater at 6 months compared with 13 months after the stoke (Figure 6A). These brain regions were repeatedly activated in many language- related cognitive tasks (Paulesu et al., 1993; Fiez et al., 1996; Smith et al., 1998). In contrast, activity in the left SPL (*x* = −20, *y* = −66, *z* = 66; *t* = 3.60, cluster size = 10, *p* svc < 0.05) was significantly greater at 13 months compared with 6 months after her stroke (Figure 6B). Activity in the left SPL was observed in the previous functional imaging studies of mental calculation tasks in abacus experts (Hanakawa et al., 2003; Chen et al., 2006; Wu et al., 2009). These functional MRI results were very consistent with the patient’s subjective report that she was able to shift the calculation strategy from a phonological – based to a mental abacus – based strategy according to her level of recovery from the stroke.

#### Digit memory task

**Figure 7:**
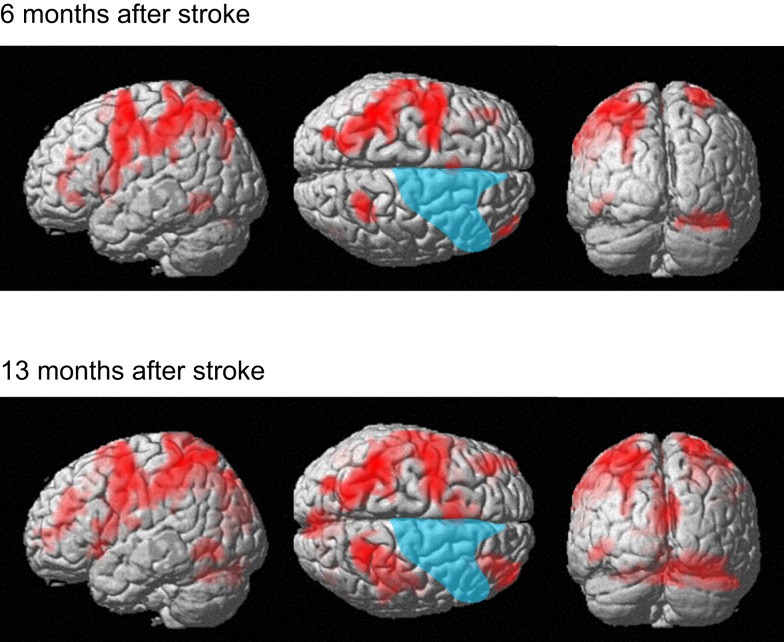
**Active areas during the delay period on the digit memory task relative to these during the visual fixation condition 6 and 13 months after her stroke**. The blue region indicates the lesion area. Brain activity is overlayed on the spatially normalized rendering images (voxel level, *p* < 0.001 uncorrected for multiple comparison; cluster level *p* < 0.05 FWE). The detailed coordinates and statistical values are listed in Table A2 in Appendix online.

The patient correctly answered all trials in both functional MRI sessions. The present analysis of the digit memory task focuses on the brain activities associated with memory retention and thus the brain activities only during the delay period are reported. Figure 7 shows brain activity associated with the delay interval period during the digit memory tasks relative to the visual fixation condition (see Table A2 in Appendix online). Overall, brain activity was left lateralized 6 months after her stroke, whereas bilateral activation was observed 13 months after her stroke. These brain regions include the inferior and middle frontal gyrus, insula, supplementary motor area, IPL, SPL, cuneus, fusiform gyrus, inferior temporal gyrus, and cerebellum.

**Figure 8:**
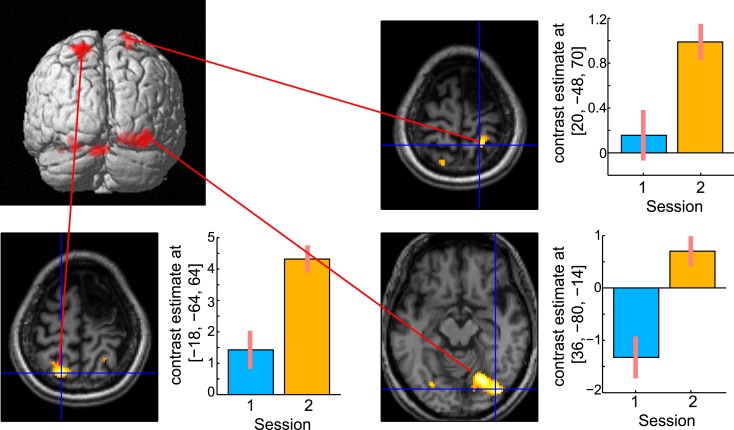
**Active areas during the retention period in the digit memory task specific to 13 months after her stroke**. The bilateral superior parietal lobule and visual association cortex were significantly activated. Statistical threshold was the same as with the mental calculation task.

A direct comparison of the brain activities observed during the delay period between the two sessions is shown in Figure 8. No brain regions were observed that showed significant regional-specific activities at 6 months compared with those at 13 months after her stroke. In contrast, activities in the bilateral SPL (left *x* = −18, *y* = −64, *z* = 64; *t* = 6.36, cluster size = 223, *p* corr < 0.05; right *x* = 20, *y* = −48, *z* = 70; *t* = 4.93, cluster size = 132, *p* corr < 0.05) and the right visual association cortex (*x* = 36, *y* = −80, *z* = −14; *t* = 6.77, cluster size = 529, *p* corr < 0.05) were significantly greater at 13 months compared with 6 months after her stroke. The bilateral activities in the SPL during the delay period were observed in the previous functional MRI study of abacus experts (Tanaka et al., 2002). Thus, the result suggests that the visuospatial strategy of mental abacus representation might be more dominantly used in the digit memory task, the same as in the mental calculation task, at 13 months after her stroke. Again, this was consistent with the patient’s subjective report that she was able to utilize the mental abacus strategy 13 months after her stroke.

#### Verbal fluency and hand grip tasks

**Figure 9:**
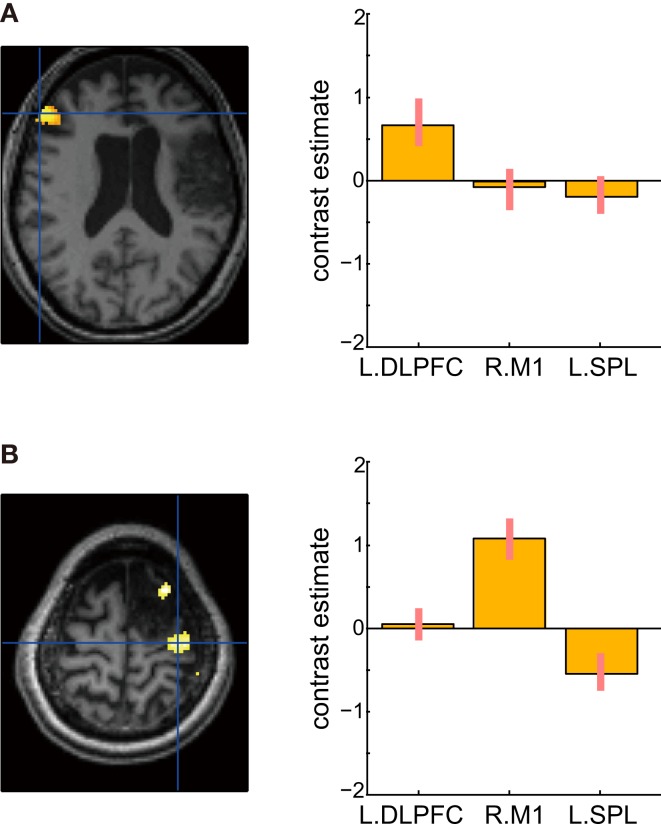
**Active areas during verbal fluency and left hand grip tasks 13 months after the stroke**. **(A)** There were significant task-specific activities in the left dorsolateral prefrontal cortex (DLPFC) for verbal fluency task and **(B)** right primary motor cortex (M1) for left hand grip task. The left SPL was not significantly activated. In each bar graph, the vertical axis indicates contrast estimated values relative to the visual fixation task, whereas the horizontal axis indicates each brain region. The error bars represent standard deviations across scans.

Figure 9 shows the results of verbal fluency and hand grip tasks. There was significant task-specific activity mainly in the left DLPFC for the verbal fluency task and in the right primary motor cortex for the left hand grip task, respectively. In contrast, in both tasks, the left SPL, which was dominantly activated during her mental calculation and digit memory tasks, was not significantly activated compared with the visual fixation condition. These findings suggest that activation in the SPL was specific to mental calculation and digit memory tasks 13 months after the stroke.

### Experiment 2

#### Behavioral experiment

**Figure 10:**
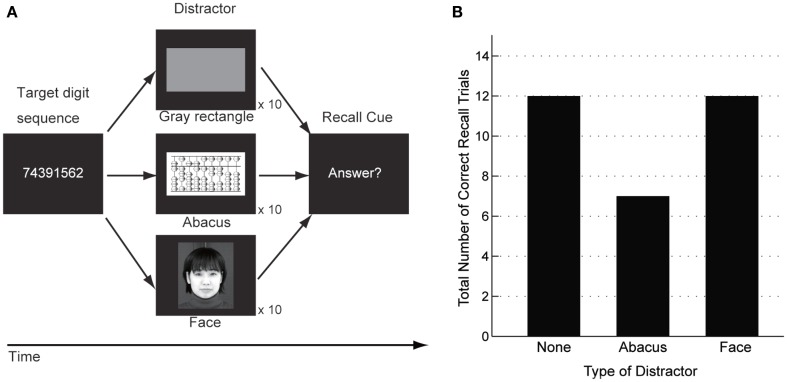
**(A)** A delayed digit recall task using interference paradigms, based on Hatta et al. (1989). The patient was asked to recall a target digit sequence after a 15-s retention interval. Three different types of visual distractors were presented during the retention interval (pictures of abacus figures, human faces, or gray rectangles). **(B)** Behavioral performance in the delayed digit recall task. The number of correctly answered trials was 12 when a human face or a gray rectangle were presented as distractors. In contrast, the number of correctly answered trials was six when the distractor was pictures of abacus figures. This result indicated that the presentation of abacus figures interfered with the patient’s digit memory performance.

The number of correctly answered trials was 12 for the human face and gray rectangle conditions, compared with 6 for the abacus picture condition (Figure 10B). Therefore, the number of the correct trials in the abacus picture condition was clearly fewer than that in the other two distractor conditions. This result showed that the presentation of pictures of abacus figures interfered with the patient’s task performance, suggesting her use of a mental abacus on the digit memory task and mental calculations 13 months after her stroke.

## Discussion

This is the first case report on the impairment of mental abacus ability by a brain lesion and on recovery-related brain activity. The patient’s knowledge and operation of basic arithmetic facts and of a physical abacus were all intact. Her impairment of arithmetic ability was specific to mental calculation and digit memory only based on the mental abacus strategy. Therefore, we consider that this would be a specific case of spatial acalculia (Hécaen et al., 1961; Hartje, 1987; Granà et al., 2006). This is a quite rare case and we have named this “abacus-based acalculia.”

The results of the present study show that brain activity during mental calculation at 13 months after her stroke was observed more in an area implicated in visuospatial working memory (Jonides et al., 1993; Mellet et al., 1996; Courtney et al., 1998a,b; Rowe et al., 2001; Tanaka et al., 2005; Oshio et al., 2010), whereas at 6 months after her stroke, brain activity was more predominant in the left hemisphere in areas related to verbal working memory (Paulesu et al., 1993; Fiez et al., 1996; Smith et al., 1998). Brain activity at 13 months after her stroke was observed in the left SPL, whereas that at 6 months after her stroke was observed in Broca’s area and the left DLPFC and IPL. This shift of region-specific brain activities is consistent with her subjective report that she was able to shift her calculation strategy from a verbal to a visuospatial strategy according to the level of her recovery from the stroke. In a behavioral experiment using interference paradigms, a visual presentation of an abacus picture, but not a human face picture, interfered with her performance of digit memory, confirming her use of the mental abacus 13 months after her stroke.

The present result is consistent with previous functional imaging studies that reported activation in the SPL during mental calculation and digit memory tasks in abacus users (Tanaka et al., 2002; Hanakawa et al., 2003; Chen et al., 2006; Wu et al., 2009). It is possible that a spatial representation of numbers is developed through abacus practice, which involves rule-based visuo motor processing, and utilized in mental calculation and digit memory tasks, because it is more efficient to mentally manipulate large numbers using a spatial representation than a sequentially organized phonological representation (Hatano et al., 1977; Hatano and Osawa, 1983 Hatano et al., 1987; Hatta et al., 1989; Hishitani, 1990; Tanaka et al., 2008; Frank and Barner, 2012). The SPL might be a key brain region for such non-verbal visuospatial representation of numbers.

According to the structural MRI, the lesion area involved the right fronto-parietal regions. Her impairment of mental abacus ability due to her right hemispheric lesion was consistent with previous behavioral and neuroimaging studies that indicate involvement of the right hemisphere in the superior arithmetic abilities of abacus users (Hatta and Ikeda, 1988; Tanaka et al., 2002; Hanakawa et al., 2003; Chen et al., 2006; Wu et al., 2009). More specifically, her lesion area included the right PMd and IPL, which have been repeatedly activated in the previous functional neuroimaging studies of abacus users (Tanaka et al., 2002; Hanakawa et al., 2003; Chen et al., 2006; Wu et al., 2009). Therefore, the present study may suggest the functional relevance of these brain regions to the mental calculation and digit memory of abacus users. However, we should be careful about such interpretations because the lesion area not only covered the PMd and IPL but also included relatively large areas of the right frontal and parietal cortex. A non-invasive brain stimulation study or neuropsychological study of patients with a more focal brain lesion will clarify this issue.

The activation in the SPL was less evident at 6 months compared with 13 months after her stroke. This implies that the damaged regions in the right hemisphere, possibly the PMd and IPL, and the SPL may work as a functional network during abacus-based mental calculation and digit memory. In fact, it is known that there is an anatomical and functional connectivity between the premotor and parietal cortex (Wise et al., 1997; Luppino et al., 1999; Wise and Murray, 2000; Tanaka et al., 2005; Oshio et al., 2010). Damage in one cortical node may induce less activity in another cortical node within the functional network. However, 13 months after her stroke, the SPL might be able to work without the damaged brain regions, possibly because of remote cortical reorganization that may occur within the intact SPL region (Frost et al., 2003; Fridman et al., 2004; Dancause et al., 2006).

In the present study, the significant activity in the SPL was left lateralized in the mental calculation task, whereas bilateral activation was found in the digit memory task. This might be due to differences in task difficulty between the two tasks, based on her subjective report after the experiment. A previous functional MRI study has reported that bilateral SPL activity in abacus users was more evident in the tasks with a higher cognitive demand (Hanakawa et al., 2003). In fact, if a lower statistical threshold was used in the mental calculation task, activation in the bilateral SPL was observed.

Regarding the task-specific activity of the SPL, one might argue that the observed differences in SPL activity among arithmetic and other control tasks (such as verbal fluency and hand grip) might be explained by the difference in task difficulty. However, that would be unlikely because the SPL activity during verbal fluency and hand grip tasks was not significantly different compared with the easiest visual fixation condition in which the subject simply watched the fixation on the screen. If the explanation of activity difference by task difficulty is true, then SPL activity during the verbal fluency and hand grip tasks should be greater than during the visual fixation task. Therefore, it is reasonable to consider that the SPL activity would be specific for her mental abacus use after her stroke recovery.

It has been proposed that the human capacity for mathematical intuition depends on both linguistic competence and visuospatial representations (Dehaene et al., 1999). By a combination of neuropsychological and neuroimaging techniques, the present finding provides evidence for an important role of visual imagery in mental arithmetic operations and also for its underlying neural correlates, the superior parietal cortex. The SPL might be an important cortical structure for non-verbal forms of number representation for calculation. The present finding may contribute to developing our understanding of the relationship between mental imagery and mental arithmetic operations.

There are several limitations for this study. First, this is a single case study and it is difficult to generalize this finding to other populations. Second, the patient was left handed and thus it is difficult to discuss the lateralization of brain activation. For this reason, we did not make any conclusions on the lateralization of brain activity from the present study. Third, the results of the behavioral interference task might be explained by a potential difference in difficulty between the distractors, such as the difference in the visual complexity of stimuli. Thus, in future studies, interference tasks should be matched for difficulty and the subject should be asked to make a behavioral response to the interfering stimuli, to be certain that the subject is actually processing the stimuli. Despite these limitations, however, we believe that this result has important implications regarding the neural substrates underlying the superior arithmetic ability of abacus users, because this is the first neuropsychological case report and also the first longitudinal functional MRI study of abacus users.

In conclusion, the present study reports for the first time a case of “abacus-based acalculia” caused by a brain lesion. Together with previous neuroimaging studies, the present result provides evidence for an important role of the PMd and parietal cortex in the mental calculation and digit memory tasks of abacus users.

## Conflict of Interest Statement

The authors declare that the research was conducted in the absence of any commercial or financial relationships that could be construed as a potential conflict of interest.

## Appendix

**Table A1:** **Brain activity during mental calculation task**.

Cluster size	Voxel *T*	MNI coordinate			Laterality	Anatomy
		*x*	*y*	*z* (mm)
**ONE-DIGIT MENTAL CALCULATION > VISUAL FIXATION 6 MONTHS AFTER THE STROKE**						
952	6.17	−42	−40	68	L	Postcentral gyrus
169	5.36	−62	−2	36	L	Postcentral gyrus
124	5.02	−64	−22	40	L	Supramarginal gyrus
108	5.00	14	−72	−44	R	Cerebellum (lobule VIIb)
178	4.86	−30	42	−10	L	Middle orbital gyrus
176	4.67	−46	0	10	L	Superior frontal gyrus
90	4.59	18	62	24	R	Superior frontal gyrus
163	4.19	−22	−4	74	L	Cerebellum (lobule VIIb)
**TWO-DIGIT MENTAL CALCULATION > VISUAL FIXATION 6 MONTHS AFTER THE STROKE**						
6452	9.27	−42	−42	68	L	Postcentral gyrus
299	7.32	16	−74	−48	R	Cerebellum (lobule VIIb)
497	6.15	26	−72	−18	R	Cerebellum
312	4.91	−22	−98	10	L	Middle occipital gyrus
1015	5.42	42	−38	62	R	Postcentral gyrus
134	4.28	−46	−64	−8	L	Inferior temporal gyrus
427	4.12	34	44	34	R	Middle frontal gyrus
**TWO-DIGIT > ONE-DIGIT MENTAL CALCULATION 6 MONTHS AFTER THE STROKE**						
290	6.42	−44	52	10	L	Middle frontal gyrus
**ONE-DIGIT MENTAL CALCULATION > VISUAL FIXATION 13 MONTHS AFTER THE STROKE**						
225	7.14	14	−74	−42	R	Cerebellum (lobule VIIb)
175	4.70	24	−70	−12	R	Fusiform gyrus
**TWO-DIGIT MENTAL CALCULATION > VISUAL FIXATION 13 MONTHS AFTER THE STROKE**						
3494	10.06	14	−74	−42	R	Cerebellum (lobule VIIb)
588	7.91	18	−56	78	R	Superior parietal lobule
1417	7.66	−20	−66	64	L	Superior parietal lobule
320	6.80	−50	−4	58	L	Precentral gyrus
581	6.42	−2	−48	−14	L	Cerebellum (vermis)
547	5.43	−22	−90	12	L	Middle occipital gyrus
266	5.73	−60	2	38	L	Precentral gyrus
136	5.51	32	−36	74	R	Postcentral gyrus
172	4.91	38	−24	48	R	Postcentral gyrus
300	4.73	40	58	4	R	Middle frontal gyrus
337	4.55	22	−98	10	R	Superior occipital gyrus
**TWO-DIGIT > ONE-DIGIT MENTAL CALCULATION 13 MONTHS AFTER THE STROKE**						
461	6.04	−18	−64	64	L	Superior parietal lobule
414	6.00	16	−60	78	R	Superior parietal lobule
95	4.59	30	−34	74	R	Postcentral gyrus
508	4.31	30	−84	10	R	Middle occipital gyrus
182	4.20	42	56	8	R	Middle frontal gyrus

**Table A2:** **Brain activity during digit memory task**.

Cluster size (number of voxel)	*T* value	MNI coordinates			Laterality	Anatomy
		*x*	*y*	*z* (mm)
**DELAY PERIOD DURING DIGIT MEMORY TASK > VISUAL FIXATION 6 MONTHS AFTER STROKE**						
7021	10.15	−62	−20	38	L	Supramarginal gyrus
656	7.97	26	−70	−20	R	Cerebellum (lobule VI)
432	6.89	26	−52	72	R	Superior parietal lobule
368	6.08	−38	34	−4	L	Inferior frontal gyrus
162	5.42	−2	12	58	L	SMA
215	4.68	−48	−54	−12	R	Inferior temporal gyrus
166	4.75	46	52	4	L	Middle frontal gyrus
178	4.7	−28	14	10	L	Insula
**DELAY PERIOD DURING DIGIT MEMORY TASK > VISUAL FIXATION 13 MONTHS AFTER STROKE**						
10518	18.9	−42	−44	56	L	Inferior parietal lobule
3206	17.87	24	−72	−16	R	Fusiform gyrus
2988	13.06	22	−50	74	R	Superior parietal lobule
495	10.04	−40	−62	−2	L	Middle temporal gyrus
946	8.67	−44	42	22	L	Middle frontal gyrus
1382	8.24	40	54	4	R	Middle frontal gyrus
581	7.75	−26	−66	−24	L	Cerebellum (lobule VI)
1552	7.62	2	−88	20	R	Cuneus
98	3.88	52	−8	20	R	Rolandic operculum

*MNI coordinates (*x, y, z*) and statistical *t*-values at the peak anatomical voxel and size of cluster (number of voxels) are listed (*P* < 0.001 uncorrected for multiple comparisons at voxel level, *p* < 0.05 corrected for multiple comparisons with Gaussian random field theory at cluster level). L indicates the left hemisphere whereas R indicates the right hemisphere*.
